# Severe Rectal Stenosis as the First Clinical Appearance of a Metastasis Originating from the Bladder: A Case Report and Literature Review

**DOI:** 10.3390/life15050682

**Published:** 2025-04-22

**Authors:** Claudiu Daha, Eugen Brătucu, Ioan Burlănescu, Virgiliu-Mihail Prunoiu, Hortensia-Alina Moisă, Ștefania Ariana Neicu, Laurențiu Simion

**Affiliations:** 1 Clinical Department No 10, General Surgery, University of Medicine and Pharmacy “Carol Davila”, 050474 Bucharest, Romania; claudiu.daha@umfcd.ro (C.D.); lasimion@yahoo.com (L.S.); 2 Department I of Oncological Surgery, Oncological Institute “Prof. Dr. Alexandru Trestioreanu”, 022328 Bucharest, Romania; 3 Department of Pathological Anatomy, Oncological Institute “Prof. Dr. Alexandru Trestioreanu”, 022328 Bucharest, Romania

**Keywords:** rare rectal metastasis, circumferential thickening, severe stenosis, difficult diagnosis, urothelial carcinoma, signet ring cell, case report

## Abstract

While locally advanced rectal cancer is the first clinical suspicion for severe rectal stenosis, in extremely unusual cases a lower bowel obstruction may be related to bladder metastasis. We present the case of a 64-year-old male who was admitted for occlusive rectal tumor (4 cm from the anal verge), for which an emergency loop-colostomy was performed. After two inconclusive endoscopic biopsies, a transanal rectal tru-cut biopsy allowed for the detection of high-grade urothelial carcinoma with signet ring cells. Furthermore, primary origin was detected in a small bladder tumor. In imaging reassessment after neoadjuvant chemotherapy, regression of the lesions both from the bladder and rectum was observed. Radical surgery with total pelvic exenteration was considered in the absence of other secondary tumors, but the patient declined and continued with radiotherapy. Subsequently he developed malignant chylous ascites and unfortunately died three months later. Reviewing the literature, we found twenty-five cases of urothelial metastasis to the rectum, originating from the bladder, including this newly present case. Rectal metastasis of urothelial origin poses a two-fold challenge in terms of both diagnosis and treatment. Determining the specific features of this uncommon manifestation of a common disease will improve future approaches.

## 1. Introduction

According to the latest statistics of the Global Cancer Observatory 2022 (GLOBOCAN), a concerning increase in bladder cancer cases has been observed worldwide, and this now ranks ninth in terms of incidence and thirteenth in mortality. Aligning with global trends Europe holds the first place for incidence, followed by Asia, which has a higher mortality rate. In Romania, bladder cancer was one the five most common cancers for both sexes in 2022, with a high incidence in men (7.1%), ranking fourth after prostate, lungs and colorectal. In the same context but placed in the foreground is colorectal cancer, which ranked first in incidence and second in terms of mortality [1]. Urinary bladder cancer preponderantly affects male patients with the mean age at diagnosis around 70 yearse, smokers, or those with a history of occupational exposure [2]. In terms of its gender distribution, women are less predisposed, with a 3:1 male/female ratio, but interestingly they are more often diagnosed with late-stage disease [2,3]. Secondary rectal lesions originating in the bladder are a rare presence in the background of these two widespread diseases. To date, only twenty-five cases have been described in the literature, including the present case. Although rectal stenosis is not part of the clinical picture specific to bladder cancer, it can, though very rarely, be the first manifestation of metastasis. Differential diagnosis of secondary colorectal neoplastic lesions remains a challenge in current endoscopic practice, due to their scarcity and clinical appearance, which may resemble a primary disorder.

Transitional cell carcinoma (TCC), also known as urothelial cell carcinoma (UCC), comprises the majority of bladder malignancies and may appear with numerous different subtypes or with divergent morphology, for example “signet ring cells”, as in the case of our patient. This occurs sporadically in 0.12–0.6% off all bladder cancers. Pure glandular differentiation is even more rare and is often difficult to distinguish from gastrointestinal adenocarcinomas or poorly differentiated prostate adenocarcinoma, without the use of immunohistochemistry [4]. A GATA 3 (nuclear marker with expression in many epithelial neoplasms) immunohistochemical staining has been shown not only to be highly specific for urothelial carcinoma, but also serves as a prognostic factor for lymphovascular and muscle invasion. In cases of moderate to strong expression, there is more likely to be no lymphovascular invasion and there is an association with low-grade or non-invasive carcinomas [5]. CDX2 positivity was initially thought to be specific to colorectal adenocarcinoma, but more recent evidence has indicated its expression in bladder cancer. However, when CDX2 staining is lacking in tumor cells, one can exclude an enteric primary site [6].

Once detected, rectal metastases from bladder cancer are not associated with a favorable prognosis. In this regard, their location does not matter as much as their late discovery. For those with single rectal metastatic localizations, no specific recommendations have been provided in the relevant guidelines to date [7,8,9]. When it comes to deciding between a radical or palliative approach, ECOG performance status and survival prognostics must be carefully evaluated and explained to the patient. The first preferred treatment in urinary bladder metastatic disease is a systemic one, usually gemcitabine-cisplatin (GC) [10]. Surgical approach may include palliative/simple loop colostomy or a radical treatment, such as pelvic exenteration or salvage surgery, as long as it improves survival and the patient’s quality of life. Selection and timing require a multidisciplinary cancer team. Through reporting the management of this new case and summarizing the previous literature, we hope to help in identifying specific features of this rare pathology, potentially enabling better diagnosis and treatment in the future.

## 2. Materials and Methods

### 2.1. Ethics Approval and Informed Consent

Written informed consent was obtained for anonymized information from medical records of the patient to be used in this research, published in accordance with our institution’s standards.

The study, in all its components, was analyzed by the Ethics Committee of the Bucharest Oncological Institute, who found that it corresponds to current ethical principles. The study does not include any research carried out on laboratory animals. The research conducted on humans is non-interventional and consists of the collection of medical data, respecting the principles of the Declaration of Helsinki.

### 2.2. Immunohistochemistry

First the hematoxylin and eosin (H and E) stain protocol was used for the study of histochemical tissue pathology.

Then, immunohistochemical stains were performed on the automated staining platform BenchMark ULTRA, produced by Ventana Medical Systems Inc., Tucson, USA. An L50-823 clone (Ventana, Roche) was used for GATA3, and an SP33 clone (Ventana, Roche) for CK20 antibody. A brightfield Leica DM2500 microscope was used to capture the microscopic images.

### 2.3. Literature Search

An up to date (1 February 2025) literature search was performed through Medline/PubMed, Scopus, Embase and Google Scholar databases using the following terms: “rectal metastasis”, “bladder cancer”, “urothelial metastasis”, “metastasis to the rectum”. About 300 relevant publications were primarily identified. We excluded metastases from the colorectum to the bladder, and others of urothelial primary origin aside from the bladder, regarding rectal metastasis. We also eliminated those studies concerning metastasis from the bladder to a location other than the rectum within the digestive system, and those with direct invasion. All articles written in English were included, as well as one written in Spanish (which was translated). One article was excluded as it did not clearly specify the number of metastases originating from the bladder and these were described collectively with those arising from the prostate. We also retrieved articles from references that treated the same subject. In total, twenty-five cases of rectal metastasis (including the one described here) were found to be eligible for secondary rectal lesions originating from the bladder, and these were further analyzed.

## 3. Detailed Case Description

A 64-year-old male, with no significant medical history, except for smoking and grade 2 hypertension, was admitted to our department for transit disorders (alternating constipation with small volume diarrhea), rectal tenesmus, moderate dysuria and significant weight loss of 6 kg over the past month. The patient was initially investigated in another urology department, where ultrasonography revealed thickened bladder walls, up to one centimeter, and right moderate hydronephrosis. A subsequent computed tomography (CT) scan of the abdomen and pelvis detected a slightly irregular circumferential parietal thickening of the middle and lower rectum, associated with mesorectal fat densification. He was first proposed for cystoscopy, but the patient declined and decided to undergo a full evaluation in our surgical oncology department.

On arrival, the physical examination revealed a slightly enlarged abdomen, but no peritonitis. In addition, digital rectal examination (DRE) found a soft velvety budded non-bleeding tumor, at four cm from the anal verge, superior to which circumferential tight stenosis could not be overcome. There were no traces of mucus or stools in the rectal ampulla, and the external anal sphincter presented a normal tone.

**Figure 1:**
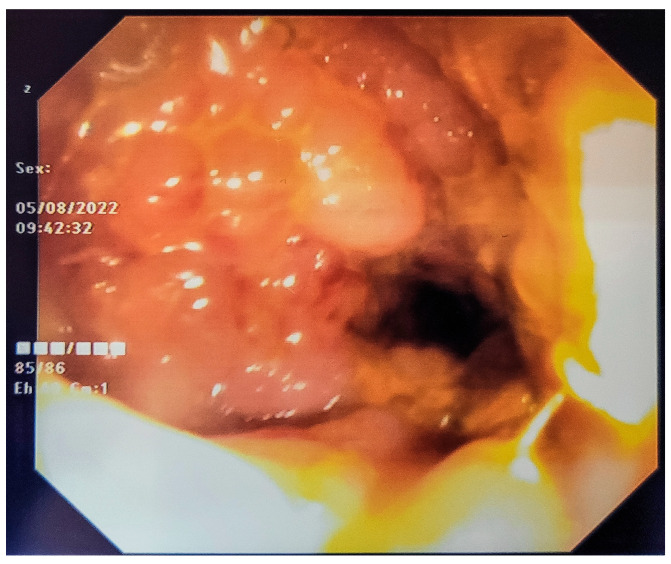
Initial rectoscopy: mucosa with” raspberry” appearance.

His laboratory values were normal for blood urea nitrogen and serum creatinine, with mild anemia. The baseline level of serum carcinoembryonic antigen (CEA) was within the normal range, while CA 19-9 was moderately increased. Given his older age and medical history, the obtained clinical-imaging findings most likely suggested a locally advanced primary rectal tumor. The patient had further undergone rectoscopy. Despite the strong likelihood of rectal cancer, a peculiar endoscopic appearance with “raspberry”-looking mucosa in the absence of rectal bleeding was considered highly atypical (Figure 1). Six biopsy samples were taken for histopathological examination. Complete colonic evaluation failed due to complete rectal stenosis 4 cm from the anal verge that could not be surpassed.

**Figure 2:**
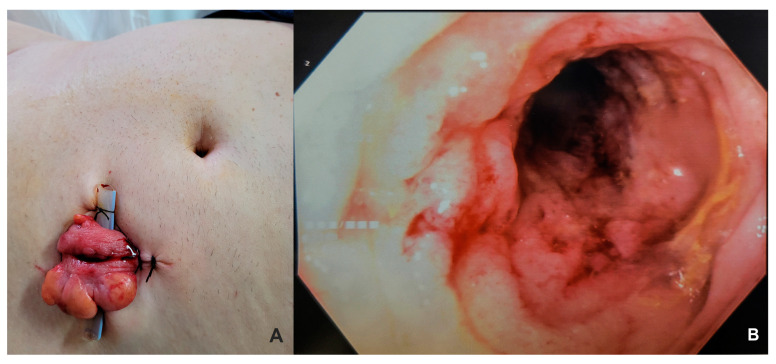
(**A**) Loop colostomy at third day after surgery; (**B**) transtomal anterograde endoscopic approach and mucosa appearance.

While waiting for histopathological conclusion, an emergency loop colostomy was performed due to a complete bowel obstruction (Figure 2A). His postoperative course was uneventful, and he was discharged on postoperative day seven.

However, the real diagnostic dilemmas began with a negative mucosal biopsy for malignancy. Reconsidering the differential diagnosis, rectal lymphoma was suspected, and iterative biopsies were taken through an anterograde transtomal approach (Figure 2B); however, similar histological findings revealed mucosa with marked edema and chronic inflammatory infiltrate, without tumor cells.

**Figure 3:**
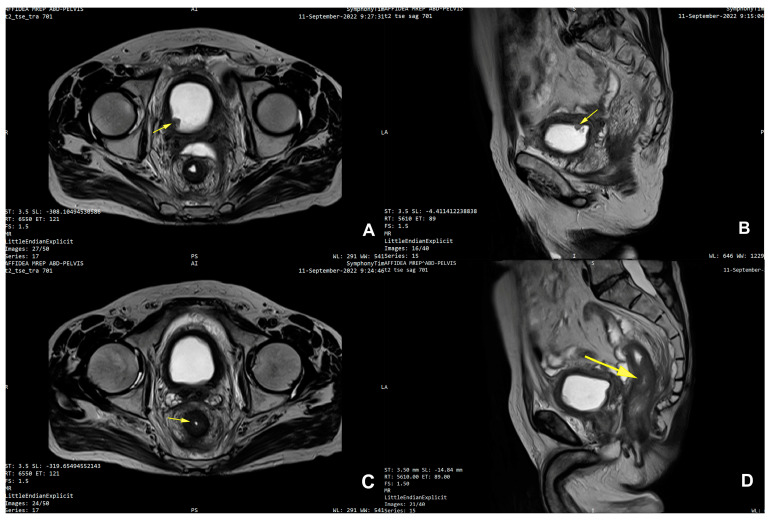
(**A**) Axial MRI diagnostic images with diffuse thickening of urinary bladder wall, non-contiguous with rectal tumor and arrow pointingthe small bladder tumor on the right postero-lateral wall; (**B**) sagittal MRI diagnostic images with arrow pointingthe bladder tumor; (**C**) axial MRI diagnostic images with arrow pointing the circumferential rectal thickening and concentric luminal stenosis; (**D**) sagittal MRI diagnostic images with arrow pointing the rectal tumoral stenosis.

Furthermore, contemporary MRI examination revealed an unexpected but small tumor on the right postero-lateral wall of the urinary bladder measuring 12/10 mm, which restricted diffusion and captured the contrasting substance (Figure 3A–D).

**Figure 4:**
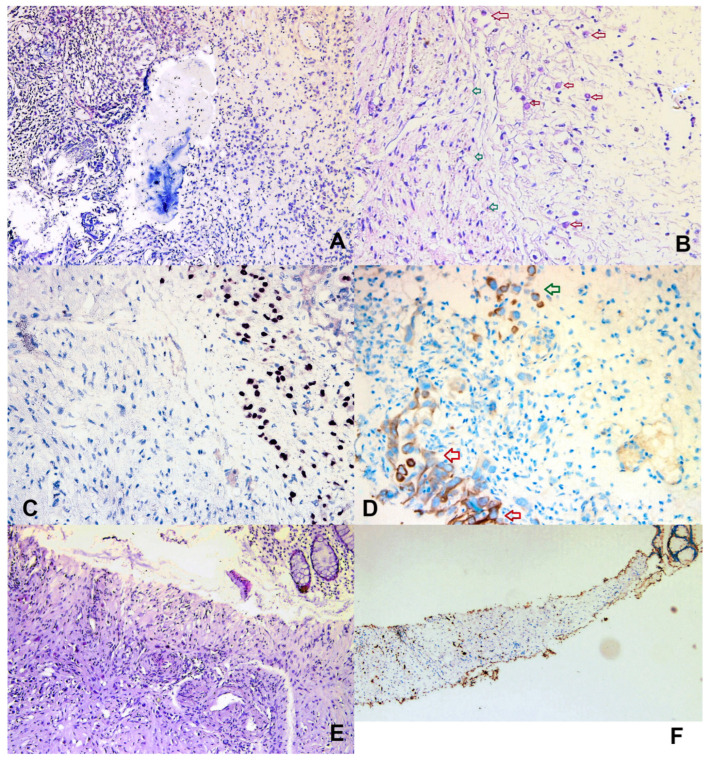
(**A**) HE stain, 100×magnification. Urinary bladder with carcinomatous infiltration: on the left, high-grade conventional urothelial carcinoma component, on the right urothelial carcinoma with foci of glandular differentiation—signet ring cells; (**B**) HE (hematoxylin and eosin) stain, 200× magnification, showing signet ring tumor cells (red arrows) and bladder muscularis propria (green arrows); (**C**) immunohistochemical staining for GATA3 in the bladder, 200× magnification: left side bladder muscularis propria, right side: invasive tumor cells; (**D**) immunohistochemical staining for CK20 in the bladder, 200× magnification: positive expression in carcinoma in situ (red arrows) and invasive signet ring cells (green arrows); (**E**) HE stain, 200× magnification, rectal biopsy: on the right corner is the colonic mucosa; in the lower half, infiltration of urothelial carcinoma diffuse variant, with signet ring cells, in the muscularis mucosae and submucosa; (**F**) immunohistochemical staining for GATA3 on rectal biopsy, 100× magnification: positive expression in tumor cells (left half of the field), suggestive of urinary tract origin of metastatic tumor cells; right corner colonic mucosa.

After the diagnostic impasse, the patient consented to more invasive procedures, such as cystoscopy, TURBT (transurethral resection of the bladder tumor) and rectal tru-cut biopsy. The transanal echo-guided approach proved to be definitive for the diagnosis. The cystoscopic findings for the corresponding imaging tumor were unexpected, with only small pseudopolypoid lesions of mucosa. Despite that, the summary results of the biopsies revealed: bladder wall fragments with infiltrative muscle invasive urothelial carcinoma (MIBC), associated with focal glandular differentiation, without perineural or lymphovascular invasion; and rectal wall with submucosal carcinomatous infiltration, providing a suggestive appearance for metastasis; furthermore, the rectal mucosa showed only chronic inflammatory infiltrate and was free of neoplasia. Immunohistochemistry (IHC) results revealed bladder biopsy with a high-grade muscle invasive urothelial carcinoma with areas of conventional and signet ring cell morphology: GATA 3 positive diffuse in tumor cells, CK 20 focal positive in tumor cells, CK5/6, P63 and CDX2 negative in tumor cells; rectal wall biopsy with same characteristics for tumor cells but with positive internal control markers for CK 20, CDX2 in colonic epithelium(Figure 4A–F).

**Figure 5:**
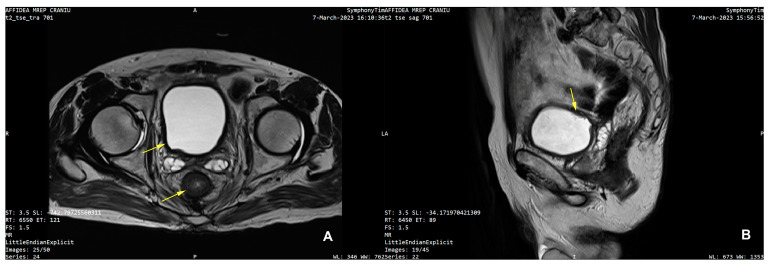
MRI re-evaluation post-chemotherapy (**A**) axial images:with arrows pointing regression of the bladder wall thickening and of the concentric rectal tumor; (**B**) sagittal view with arrow pointing downsized bladder tumor.

The case was discussed in the tumor board and referred to neoadjuvant chemotherapy. Systemic treatment with gemcitabine–cisplatin (GC) led to encouraging results after completing six cycles. Imagistic re-evaluation showed tumor regression both at the level of the bladder (parietal thickening of 4 mm compared to 7 mm previously, tumor size decrease from 12/10 mm to 4/5 mm, and moderate capture of the contrast substance, prostate with integral capsule) and rectum (slight thickening of the right postero-lateral wall of 15 mm compared to 25 mm previously, with restricted diffusion and no other new secondary lesions or free peritoneal fluid) (Figure 5 A,B). In the absence of distant metastases the patient was proposed for pelvic exenteration.

The patient refused complex surgery, with higher risk implying an additional stoma, thus he was proposed for radiotherapy. External beam radiation therapy targeting the bladder, mesorectum and regional lymph nodes was even more difficult to bear, but he completed a total dose of 56 Gy of 60 Gy initially proposed, due to bone marrow suppression (thrombocytopenia). During this period, the patient also had an episode of diarrhea, likely secondary to mucositis.

**Figure 6:**
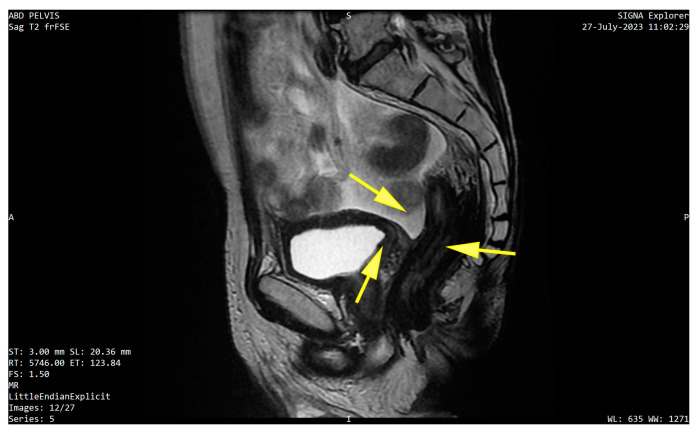
MRI sagittal images after chemoradiotherapy with arrows pointing: ascites in the rectovesical pouch and thickening of the bladder and rectal wall.

However, the patient’s health status remained poor, with significant additional weight loss and anorexia. The subsequent MRI revealed no evidence of new metastases, rectal changes that were rather fibrotic, and at the level of the bladder a persistent area which captured contrast (Figure 6). Cystoscopic re-evaluation evidenced an ulcerated area on the posterior wall about 2 cm in diameter and diagnostic TURBT was scheduled three days later.

Unfortunately, intraperitoneal perforation occurred during the procedure, due to thinness of the bladder wall. At the time of perforation, an intensely turbid liquid with a chylous-like appearance in a large quantity (approx. 1000 mL) originating from the peritoneal cavity flooded the bladder unexpectedly.

**Figure 7:**
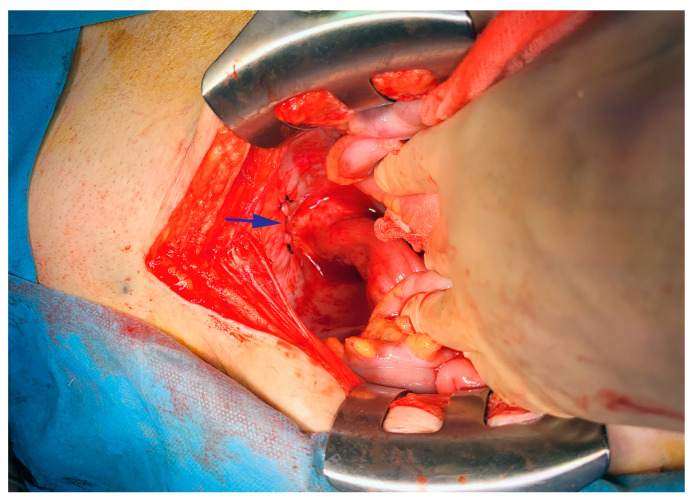
Intraoperative aspect of peritoneum with arrow pointing the bladder suture.

Emergency laparotomy was decided on and bladder suture was performed. Intraoperatively, the same aspect for the fluid was found without detecting a cause. The peritoneum had a normal appearance but there was a mild intestinal, mesenteric and mesocolic edema (Figure 7). Liquid bacteriology was negative, but malignant cells were detected through cytology. Histopathological examination of the resected bladder tumor revealed post-radiation therapy fibrosis without any malignant elements. The chylous appearance of the peritoneal drainage persisted during the postoperative period.

Although the patient had an initial good response to treatment, continuous deterioration remained unexplained, without evidence for bulky tumor or metastases. During this period, palliative care was provided until his unfortunate death three months later, at home, with significant weight loss, anorexia, and progressive ascites.

## 4. Literature Review

Solitary cases have been reported in the literature over a period of 30 years (from 1996 to 2025), except for a series of two cases reported by Yusuf et al. (2005) [11], and three cases described by Gleason (2008) [12].

The patients’ ages ranged from 53 to 85 years, with an average of 64. Male/female ratio was 7:1. Most patients already had a history of bladder cancer treatment when they were diagnosed with rectal metastasis, and only six were synchronous occurrences. The symptoms at presentation were predominantly digestive, with transit disorders, occlusive phenomena, and only rarely intestinal bleeding (Table 1) Diagnostic difficulties were frequently encountered, 9 cases reporting inconclusive colonoscopic biopsies, including present one. Imaging-based findings (MRI, CT, echo-endoscopy) had a primary role. Time interval from diagnosis of bladder cancer to detection of rectal metastasis ranged from 1 month to 10 years. A small number of patients also presented other metastases. The lower rectum was affected in almost all cases. An apparently intact mucosa in endoscopy was characteristic, with a few exceptions.

Due to the increased size of the rectal tumor and the tight stenosis in a significant number of cases, continuous colostomy was the only surgical procedure. Salvage and radical surgery were feasible only in a few cases, and did not prolong survival compared with other therapeutic approaches. Apart from colostomy, palliative treatment ranged from chemotherapy to radiotherapy and immunotherapy as well as association of these approaches. Overall survival ranged from a few days to fifteen months (in our case) (Table 2).

No specific serum tumor markers have been described, although, in a few cases (including ours), CA 19.9 was moderately elevated. The most commonly used markers for immunohistochemical confirmation of urothelial origin were cytokeratin 7 (CK 7), cytokeratin (CK 20) and GATA binding protein 3 (GATA 3) and, for the exclusion of digestive origin, caudal-related homeobox gene 2 (CDX2) was used.

## 5. Discussion

In daily clinical practice, rectal metastases originating in the bladder are an extremely rare occurrence. The case we encountered is the twenty-fifth reported so far in the world, according to the literature review conducted up to 1 February 2025.

For both bladder and rectal cancer, local bleeding is an important pathognomonic sign, which was missing for our patient. Interestingly, although visible painless hematuria is the most common clinical manifestation of bladder cancer (present in 75% of cases) [32], and although our patient was in an advanced stage of disease, with an aggressive type of tumor, the symptoms were associated with rectal metastasis rather than with the primary tumor. The symptomatology found in the literature, also predominantly digestive, may be explained by the fact that many of the patients (60%) had already undergone radical cystectomy. This explains the lack of urinary bleeding, but not digestive bleeding [33], which is due to the absence of invasion to the rectal mucosa. Only three out of the reported twenty-five patients presented with hematochezia or rectal bleeding.

Endoscopic findings were described in many cases as intact or erythematous mucosa, and only rarely with frond-like [31], cobblestone [17] or raspberry appearance, as in our case. The presence of cobblestone mucosa during rectoscopy is not pathognomonic but may suggest specific conditions, such as inflammatory bowel disease, thus requiring further diagnostic tests [34]. In a retrospective study analyzing the endoscopic aspect of forty-two gastrointestinal secondary lesions, for both lower and upper endoscopies, Wei et al. calculated a misdiagnosis rate of up to 40.5%, first by considering the lesions as primarily malignant, and secondly by considering them as benign [35]. These results indicate the difficulty of an etiological diagnosis based on endoscopy, in addition to its time-consuming nature They documented a lower incidence in the colorectal segment (9/42 patients), with a majority of rectal lesions (three out of four) originating from the bladder or prostate [35].

While most of the patients in the cohort that we reviewed had a history of bladder cancer, it took between one month and ten years to confirm the second diagnosis due to a recurrence-free interval. Therefore, prolonged closed surveillance may be needed in bladder cancer patients. Although the tumors were accessible to digital rectal examination and did not require expansive tests, paradoxically, they were detected late in the oncological follow-up of the patients.

In contrast to the apparently normal overlying mucosa, various degrees of concentric stenosis have been described in the case reports, with symptoms ranging from mild transit disorders to complete occlusion, as in our case. When diagnosing the underlying cause of rectal circumferential stenosis, one must consider a previous medical history of inflammatory strictures (which usually affects young adults with bowel benign disease) [34], trauma or healing after surgery (mechanical anastomosis, TEM surgery or hemorrhoidectomy), or even post-local radiotherapy. Other primary conditions, such as colorectal lymphomas, may affect elderly patients, with males presenting increased susceptibility, but caecum is first affected, and nodal involvement is common [36].

Another contradiction that misled us during the routine diagnostics process was the larger dimensions of the rectal secondary tumor compared with the small sized primary bladder tumor Ta-T1. In the absence of imaging contiguity between the two solid masses, our initial suspicion was of synchronous distinct neoplasia. A secondary bladder tumor from a colorectal cancer was also possible, but metastases more frequently arise from the prostate, uterus and ovaries [37,38]. Bladder cancer usually metastasizes to the lymph nodes, liver, lung and bones, and only rarely to the intestinal tract, in 13% of cases according to Yusuf et al. (11] In a multi-institutional study including seven European centers, Mourra et al. tracked only thirty-five patients with truly colorectal metastases; however, they excluded those with TCC. The first two incriminated were breast cancer and melanoma, accounting for 68.57% of all cases [39]. The most probable pathway of metastasis in these cases was hematogenous.

Apparently, a common case of rectal malignant stenosis [40,41,42,43] turned into a difficult diagnostic process that involved iterative biopsies. We performed two subsequent colonoscopic biopsies with multiple false negative pathologic reports due to the absence of rectal mucosa invasion. The next logical step in diagnosis was to change the biopsy approach towards a deeper manner, due to the thickening of the rectal wall, as confirmed by both endoscopic ultrasound (EUS) and MRI. An ultrasound-guided transrectal tru-cut biopsy was crucial in this case for diagnosis, helping to identify the area of maximum thickness with the lowest risk of perforation.

When searching the literature, echoendoscopy appears to have been proven to be equally useful for characterizing and diagnosing lesions in the rectum. In this regard, Gleeson et al. presented the largest EUS case series in search of specific features of rectal wall metastases in order to strengthen the use of EUS–FNA and EUS–TCB [12], although colonoscopic biopsies provided repeated false-negative results in many cases, ultrasound-guided and tru-cut biopsies have always been diagnostic. Active surveillance with transrectal ultrasound in patients with previous bladder cancer surgery could allow for the detection of thickening of the submucosa and recommendation of deep biopsy when suspicion is high, unmasking possible early rectal metastases. Minor symptoms may go unnoticed, probably attributed to postoperative and radiotherapy side effects; however, presentation at the first clinical changes should be encouraged.

The resumption of evolution occurred unexpectedly and suddenly. In their follow-up, the clinical picture at presentation was low occlusion, similar to that observed in our patient. At the forefront of the treatment scheme was the urgent loop-colostomy for low obstruction, a saving surgical gesture that led to a decrease in local pain and subsequently allowed the patient to be rebalanced. In advanced stages of disease, lower intestinal occlusion is typical for both primary and secondary rectal lesions. The absence of bleeding and presence of the concentric thickening with luminal narrowing may be the only difference for secondary lesions, especially transit disorders, and rectal discomfort may also be considered due to previous therapies. A linitis-like MRI aspect in the presence of normal mucosa may be a trustworthy indicator for suspicion of the deep secondary invasion of the rectum, in patients with medical history of bladder cancer [44].

Immunohistochemical studies are required to accurately specify the primary origin of neoplasia. Intratumoral histological heterogeneity is quite frequent in bladder cancer, in up to 41% of cases, with a wide spectrum of subtypes and divergent differentiation. Immunohistochemistry can help to successfully distinguish co-existing subtypes within the tumor, from pure squamous to glandular differentiation [45]. In this particular case, establishment of the neoplastic primary site was hampered by the presence of “signet ring” cells, which are typical of gastrointestinal neoplasia and rarely encountered in bladder cancer. This is the third case described in the literature of rectal metastasis originating in the bladder with a signet ring component. Poor specific signs in this new case could be attributable to the “signet ring” cell variant of urothelial carcinoma, often incriminated for submucosal infiltrative forms [46]. Colorectal adenocarcinoma is typically CDX-2 positive, cytokeratin (CK) 20 positive, and CK 7 negative. Due to great advances in the morphopathological field, we may expect an increase in the identification of such rare scenarios for which specific treatment protocols have not been yet established. Cytokeratin (CK) 7 and CK 20 were found to be positive in patients with bladder adenocarcinoma, but for colon primary origin CK 7 negativity was observed [5,6].

Different rectal dissemination pathways of the bladder cancer have been discussed in the literature, including lymphatic, hematogenous, intraoperative seeding, peritoneal routes, invasion of the Denonvilliers’ fascia and via the postero-lateral pedicles of the bladder [47].

Our patient did not have previous surgery so intraoperative seeding was excluded. CT and MRI imaging investigations did not detect other metastases, so hematogenous metastasis was improbable. No invaded lymph nodes were detected either, although a PET–CT scan would have brought additional information in this regard. As the patient declined radical surgery, we could not exclude lymphatic micrometastasis. Later in the patient’s clinical course, malignant chylous ascites were found but no obvious macroscopic peritoneal seed. With a complete pathological response of the bladder after neoadjuvant therapy (confirmed by TURB specimen), we can exclude direct tumoral invasion of the peritoneum or a possible procedural contamination at the time of bladder perforation. Despite these facts, an evident positive cytology for malignancy was found.

Intraoperatively we observed only a slight diffuse peritoneal and visceral edema and, while multiple biopsies would have been useful for a certain diagnosis, they were of little importance for the immediate therapeutic approach. The milky peritoneal fluid proved to be rich in malignant cells, which argues for the provenance of the cells from the disrupted lymphatic vessels, as well as for poor cellular cohesiveness.

All these findings led us to put forward a new lymphatic metastasis hypothesis for our patient, i.e., a possible carcinomatous lymphangitis, which could explain the chylous ascites due to massive invasion and obstruction of the lymphatic network, in the absence of obvious involvement of the lymph nodes. Carcinomatous lymphangitis is described as a particular form of lymphatic dissemination in which malignant cells invade lymphatic vessels, with or without lymph node invasion [48,49,50,51].

Non-specific symptoms, such as anorexia, that lead to progressive clinical deterioration of the patient advocate for impaired intestinal absorption and loss of nutrients due to chylous effusion. This could explain the patient’s poor outcome, although chemoradiotherapy treatment led to evident tumor regression, but thesemanifestations are usually attributed to systemic therapies masking other causes.

To the best of our knowledge, this is the first case reported with secondary chylous-type ascites, probably due to carcinomatous lymphangitis in a patient with urothelial rectal metastasis originating in the bladder. In the existing literature, we found extrapulmonary carcinomatous lymphangitis to be very rare, with only a few cases recorded [51,52]. One study on cadavers analyzed the lymphatic configuration of the bladder and rectum in males, showing a similarity in the richness of the vessels, which could explain the affinity of malignant cells [53]. Communications between the lymphatic drainage of the bladder and the rectum may also explain such metastases.

Zheng et al. recently observed how fibroblast promotes lymphovascular invasion and lymphangiogenesis, even in the early stage of urothelial bladder cancer, and described the dynamic microenvironment of tumor cells. These remarks contradict preconceived ideas, such as the passive role of lymphatic vessels, demonstrating that they are not only collecting tubes [54,55] but also active players in the metastasis process. New therapies are emerging that can block this process by targeting key receptors. The model they reproduced does not involve the postradiotherapy changes that were probably encountered in our case, nor metastasis to other neighboring organs. Not all the different hypotheses proposed regarding the metastasis route have been validated, due to the small number of cases and a lack of postmortem analyses [56,57].

Another aspect should be noted. Although the bladder cancer apparently responded well to treatment (even complete remission) in some cases, the occurrence of rectal metastasis was unexpected and completely altered patient’s prognosis. The presence of signet ring cell carcinoma (SRCC), indicating a rare type of bladder malignancy, and histological grade are decisive factors in recurrence and progression [46]. Muscle invasive bladder cancer (MIBC) is poorly differentiated in 96% of cases. Although not yet part of current clinical practice, there are ongoing studies on molecular mechanisms in bladder cancer that can be expected to lead to the discovery of new valid treatments, even in the metastatic stage [58,59]. The expression of individual biomarkers as Excision Repair Cross-Complementation 1 (ERCC1) could explain the poor response to chemotherapy in patients with metastatic bladder cancer [60].

In most cases, rectal metastasis was a solitary recurrence for the bladder cancer, but the outcome was still unfavorable.

In selected cases with metastatic disease, urothelial cell carcinoma is mostly chemo-sensitive in its ubiquitous type [61]. Regarding prognostic factors for systemic treatment, five-year survival rates were strongly negatively correlated with the existence of visceral metastases and/or Karnofsky performance status [10]. A cautious choice between platinum-based drug and platinum-free treatment may help to avoid myelosuppression and mucositis [62].

Although rectal metastases in urothelial carcinoma (UC) have a poor prognosis, in this case good control of the disease was achieved with systemic therapy [63,64,65,66] and local radiotherapy after minimal surgical intervention for rectal obstruction. The unique rectal metastasis and good response after neoadjuvant chemotherapy made the case eligible for radical pelvic exenteration, but the patient refused this due to the additional stoma. It is doubtful whether this therapeutic approach would have prolonged survival.

There was a noticeable difference in male to female ratio (7:1) in patients with rectal metastases compared with that in patients with bladder cancer without rectal metastases (3:1), which can be explained by particulars of the pelvic anatomy and lymphatic routes.

### Study Limitation

The small number of patients with rectal metastases from bladder carcinoma naturally restricts the extent of clinical data available, and this in turn limits the generalizability of the reported findings.

Furthermore, the retrospective nature of the literature review precludes prospective validation of the proposed diagnostic and therapeutic strategies.

## 6. Conclusions

Symptoms related to annular rectal stenosis may be the first manifestation of a urothelial neoplasia.

Findings regarding the etiology of a concentric thickening rectal stenosis with normal overlying mucosa obtained through traditional methods should be re-evaluated in favor of EUS tru-cut guided biopsy, enabling faster diagnosis and also providing sufficient specimens for detailed immunohistopathological analysis.

Overall survival after detecting rectal metastasis remains quite low, but in some cases systemic treatment and radiotherapy could improve the prognosis. The utility of major radical surgery is unclear.

Future in-depth studies of these particular cases are needed, in order to optimize the therapeutic approach for similarly affected patients.

## Figures and Tables

**Table 1:** Pretreatment clinical and diagnostic finding in studies reporting rectal metastases originating from the bladder.

	Author (Year)	Age/Sex	Pathology	IHC Positive/Negative	Biopsy Approach	Symptoms	Rectal Tumor Extension
**1**	Langenstroer P et al. (1996) [13]	73/M	TCC PD/focal adenocarcinoma and signet ring cell	NO	needle	fatigue, weight loss, chronic diarrhea, rectal pain	DISTAL R
**2**	Ha HK et al. (2000) [14]	N/S	TCC	NO	NS	NS	NS
**3**	Hong SP et al. (2002) [15]	63/M	TCC papillary	NO	colonoscopy	jaundice, constipation, weight loss	NS
**4**	Yusuf TE et al. (2005) [11]	54/M	TCC G3	NO	mucosal biopsy negative, EUS-FNA AND EUS-TCB	change in bowel habits	DISTAL R
**5**	Yusuf TE et al. (2005) [11]	73/M	TCC PD	NO	mucosal biopsy negative, EUS-FNA	severe constipation	DISTAL R
**6**	Gleeson FC et al. (2008) [12]	54/M	TCC/G3	CK 7, CK 20	EUS-FNA AND EUS-TCB	presumed refractory ulcerative proctitis	DISTAL MEDIUM R
**7**	Gleeson FC et al. (2008) [12]	55/M	TCC/G3	CK 7, CK 20	EUS FNA AND EUS TCB	altered bowel habit (constipation)	ALL RECTUM
**8**	Gleeson FC et al. (2008) [12]	60/M	TCC/G3	CK7, CK 20	EUS FNA	altered bowel habit (constipation)	RECTUM
**9**	Ying-Yue J et al. (2010) [16]	83/M	UCC PD	CK 7, CK 20/CDX-2 SI S-100	rectoscopy	frequent bowel movements, weight loss	DISTAL R
**10**	Katsinelos P et al. (2012) [17]	68/M	TCC G3 papillary	CK7, CK 20/PSA	multiple biopsies and FNA negative	anal outlet obstruction, constipation, tenesmus	DISTAL R
**11**	Yang M-H et al. (2012) [18]	61/M	UCC PD	CEA, CK 7, CK 20/PSA, SYNAPTOPHYSIN	colonoscopy	difficult defecation, abdominal distension	DISTAL R
**12**	Kassam et al. (2013) [19]	53/M	TCC PD	CK 7, CK20	colonoscopy	anorexia, diffuse abdominal pain, pencil thin stools	RECTOSIGMOID
**13**	Asfour R et al. (2014) [20]	54/M	UCC	NO	colonoscopy	constipation, anorexia, pain with defecation, abdominal distension	DISTAL R
**14**	Hashash JG et al. (2015) [21]	55/M	UCC	KI 67, P 53, GATA 3	rectal tunnel biopsies	rectal pain, urgency, heaviness, thinning stool	DISTAL MEDIUM R
**15**	Ma Y et al. (2015) [22]	65/M	UCC PD	P53, CK 34, CK7	colonoscopy	increasing constipation, faecal incontinence and tenesmus	DISTAL R
**16**	Esfandiari et al. (2016) [23]	84/F	UCC papillary G3	CK 7, EMA, P40, GATA3, CA 125/equivocal BerEP4	sigmoidoscopy TCB	rectal pain on defecation, suprapubic pain, fecal and urinary incontinence	MEDIUM R
**17**	Chung Sang TSE et al. (2020) [24]	85/M	UCC	CK 7, CK 20, GATA3	sigmoidoscopy	scrotal pain and swelling, hematochezia	RECTUM
**18**	Andreea de Cino et al. (2021) [25]	81/M	UCC	NO	sigmoidoscopy	subacute epigastric pain, chronic constipation	NS
**19**	Li Y et al. (2021) [26]	72/F	UCC G2	CK 7, CK 20, GATA3/CDX2	colonoscopy	abdominal bloating and obstructed defecation	DISTAL MEDIUM R
**20**	Nazar et al. (2022) [27]	72/F	UCC PD	GATA3, CK20/TTF1, CDX, P63, PAX 8	colonoscopy	hematochezia, tenesmus, rectal pain	UPPER R
**21**	Nagahisa C et al. (2023) [28]	67/M	UCC	GATA3/CDX 2	colonoscopy	severe constipation, anal pain	DISTAL R
**22**	Yang et al. (2023) [29]	64/M	UCC	KI 67, P53, CK, GATA3, CK7	colonoscopy, multiple rectal, finally EUS-FNAdiagnostic	increased frequency of defecation, decrease stool volume	DISTAL MEDIUM R
**23**	Alnasarat A et al. (2024) [30]	85/M	UCC plasmacytoid, signet ring	GATA, CK 7, CK 20/CDX	sigmoidoscopy	weight loss, epigastric discomfort, abdominal distension	RECTUM
**24**	Menon R et al. (2024) [31]	75/M	UCC	P63, GATA3	sigmoidoscopy	rectal bleeding	DISTAL R
**25**	Present Case (2025)	64/M	UCC G3/conventional and signet ring cell	GATA3, CK 20/CK5/6, P63, CDX	colonoscopy, rectal EUS TCB	rectal tenesmus, transit disorders (constipation/small volume diarrhea), moderate dysuria	DISTAL MEDIUM R

FNA: fine needle aspiration; UCC: urothelial cell carcinoma; TCC: transitional cell carcinoma; PD: poorly differentiated; IHC: immunohistochemistry; R: rectum; TCB: tru-cut biopsy; EUS: ultrasound guided endoscopy.

**Table 2:** Previous and current treatment approaches for patients with rectal metastasis from bladder cancer.

Author (Year)		Age/Sex	Time Months Till Rectal Finding	Previous Treatment for Bladder Cancer	Treatment for Rectal Metastasis	Survival Months With RM	TNM/Stage
1.	Langenstroer P et al. (1996) [13]	73/M	34	TURBT, RC WITH IC	EXPL-LAP, loop-colostomy, CHT PALLIATIVE recommended	2 M/D	pT3a N0, NSM
2.	Ha HK et al. (2000) [14]	N/S	N/S	N/S	N/S	N/S	N/S
3.	Hong SP et al. (2002) [15]	63/M	10	RC, ADJUVANT CHT MVAC	RT external and brachytherapy	4 M/A	N/S
4.	Yusuf TE et al. (2005) [11]	54/M	24	RC ILEAL NEOBLADDER	CHT	N/S	N/S
5.	Yusuf TE et al. (2005) [11]	73/M	24	SURGICALLY RESECTED	CHT TPE	N/S	N/S
6.	Gleeson FC et al. (2008) [12]	54/M	18-119	RC WITH NEOBLADDER	CHT	N/S	N/S
7.	Gleeson FC et al. (2008) [12]	55/M	18-119	RC WITH NEOBLADDER	CHT	N/S	N/S
8.	Gleeson FC et al. (2008) [12]	60/M	18-119	RC WITH NEOBLADDER	CHT	N/S	N/S
9.	Ying-Yue J et al. (2010) [16]	83/M	0	NO	CHT	N/S	N/S
10.	Katsinelos P et al. (2012) [17]	68/M	0	NO	RECTAL RESECTION AND RC WITH LND, adj RT, CHT(GC)	8 M/D	SMALL T
11.	Yang M-H et al. (2012) [18]	61/M	10	TURBT, NEOADJ CHT (PACLITAXEL, GEM, CARBO) RC WITH IC, CHT ADJ	CHT FOLFIRI shrinkage	3 M/A	T2 downstaged T0
12.	Kassam et al. (2013) [19]	53/M	17	TURBT RC WITH BP LND AND IC, ADJ CHT GC	rectal stent and PALLIATIVE CARE	6 M/D	pT3aN1M0
13.	Asfour R et al. (2014) [20]	54/M	6	TURBT, CHT MITOMYCIN C, GC X 4, RC WITH NEOBLADDER AND P LND	loop-colostomy	N/S A	N1
14.	Hashash JG et al. (2015) [21]	55/M	10	NEOADJ CHT, RC, P LND AND IC	Loop-colostomy, CHT-RT PALLIATIVE	N/S	T2-3/II
15.	Ma Y et al. (2015) [22]	65/M	23	RC WITH BP LND AND IC, ADJ CHT GC	CHT RT PALLIATIVE end-colostomy	N/S	PT3N0M0, NSM
16.	Esfandiari et al. (2016) [23]	84/F	0	NO	RT PALLIATIVE, colostomy	1 M/A	N/S
17.	Chung Sang TSE et al. (2020) [24]	85/M	96	RC AND IC	PALLIATIVE IMMUNOTHERAPY NOT TOLERATED	6 M/D	pT3aN0MO/III A
18.	Andreea de Cino et al. (2021) [25]	81/M	N/S	TURBT, CHT	third line CHT	N/S	N1
19.	Li Y et al. (2021) [26]	72/F	48	TURBT MULTIPLE, BCG X 6	LAPAROSCOPY peritoneal nodule biopsy, CHT GC, loop-colostomy	10 M/A	pTAN0M0/Oa
20.	Nazar et al. (2022) [27]	72/F	SAME YEAR	RADICAL CYSTECTOMY WITH TOTAL HYSTERECTOMY AND DOUBLE ADNEXECTOMY, CHT CARBO, GEM ADJ	PMB, eventually RT	N/S	T4N1M1 OSS
21.	Nagahisa C et al. (2023) [28]	67/M	1	TURBT, CHT GC, RC WITH BP LND AND IC	Colostomy RT PMB	10 M/A	T2N0M0/PT4aN0, NSM
22.	Yang et al. (2023) [29]	64/M	0	NO	TPE WITH IORT, ADJ CHT GC, Sintilimab	A	pT3bN2M1b
23.	Alnasarat A et al. (2024) [30]	85/M	0	NO	PALLIATIVE CARE	FEW DAYS/D	N/S
24.	Menon R et al. (2024) [31]	75/M	N/S	CHT CISPLATIN RT TURBT, RC WITH IC	EXPL LAP, colostomy	N/S	T1N0M0/I
25.	Present Case (2025)	64/M	0	NO	Colostomy, NEOADJ CHT GC, RT	15 M/D	T2N0M0

M: Male; F: Female; RC: Radical Cysto-prostatectomy; IC: ileal conduit; BP: bilateral pelvic; P: Pelvic; LND: lymph node dissection; TURBT-transurethral resection of bladder tumor; EXPL LAP: Exploratory Laparotomy; TPE: total pelvic exenteration; NSM: negative surgical margins; D: Death; A: Alive; RT: Radiotherapy; IORT: Intraoperative Radiation Therapy; CHT: Chemotherapy; BCG: Bacillus Calmette–Guerin, PMB: Pembrolizumab; ADJ: Adjuvant; NEOADJ: Neoadjuvant; N/S: not specified; GC: Gemcitabina Cisplatin; MVAC: METHOTREXATE, VINBLASTINE, DOXORUBICIN AND CISPLATIN; CARBO-Carboplatin; FOLFIRI: FOLINIC ACID, FLUOROURACIL AND IRINOTECAN.

## Data Availability

The patient’s data were obtained from the medical documents of the Bucharest Oncological Institute and cannot be made publicly available as they contain personal and confidential data of the patient, but any information about these documents can be obtained upon reasonable request from the corresponding author.
